# Major depressive disorder with anxious features - the role of pregabalin

**DOI:** 10.1192/j.eurpsy.2023.1785

**Published:** 2023-07-19

**Authors:** R. S. Carvalho, B. R. Afonso, E. Santos

**Affiliations:** Psiquiatria, Hospital de Magalhães Lemos, Porto, Portugal

## Abstract

**Introduction:**

Patients with unipolar major depression often present with symptoms of anxiety. Presentations with high levels of anxiety, restlessness, and somatic correlates of anxiety can be clinically identified as anxious depression. The comorbidity of anxious and depressive symptoms is a marker of poor prognosis, with greater risk of relapse and increased suicidal risk.

**Objectives:**

Brief review of the role of pregabalin in the treatment of major depressive disorder (MDD), based on a case study.

**Methods:**

Consultation of the clinical record and brief review of the literature on this subject.

**Results:**

We present the case of a 25-year-old woman, with no past psychiatric history, admitted to a psychiatric consultation with depressive symptoms, and marked anxiety and somatic complaints, such as restlessness, palpitations, gastrointestinal discomfort. She fulfilled diagnostic criteria for Major depressive disorder (MDD), and was initially treated with sertraline 50 mg, with partial response, but maintenance of prominent anxious symptoms with important functional impairment. Then, we raised the dose of sertraline to 100 mg and added pregabalin 50 mg, with up-titration to 150 mg per day, divided in three doses. We observed rapid response, particularly on the anxious symptoms, and subsequently on the patient functionality.

The anxiety symptoms can increase in the first days of treatment with a selective serotonin reuptake inhibitor, which is the first-line therapy for major depression. Those are particularly difficult to treat, resulting often in treatment resistance and functional impairment. Pregabalin has a proven rapid-onset anxiolytic effect, with less cognitive and motor effects and less risk for dependence than benzodiazepines. It has demonstrated efficacy for the treatment of generalized anxiety disorder, but the use for patients with MDD has not been clearly studied.

**Conclusions:**

Although the evidence on this subject is sparse, pregabalin augmentation of antidepressants could be an adequate option for the treatment of depression, allowing a faster action on the anxiety symptoms, especially on the first weeks of treatment, without some of the risks of the benzodiazepines.

**Disclosure of Interest:**

None Declared

